# Age, Body Mass Index, and Waist-to-Hip Ratio Related Changes in Insulin Secretion  and  Insulin Sensitivity in Women  with Polycystic Ovary Syndrome: Minimal Model Analyses

**DOI:** 10.1155/2022/6630498

**Published:** 2022-05-18

**Authors:** Mirjana Šumarac-Dumanović, Danica Stamenković-Pejković, Danka Jeremić, Janko Dumanović, Vesna Mandić-Marković, Miloš Žarković, Dragan Micić

**Affiliations:** ^1^School of Medicine, University of Belgrade, Belgrade, Serbia; ^2^Clinic for Endocrinology, Diabetes and Diseases of Metabolism, Clinical Center of Serbia, Belgrade, Serbia; ^3^Obstetrics and Gynaecology Clinic Narodni Front, Belgrade, Serbia; ^4^Department of Medical Sciences, Serbian Academy of Sciences and Arts, Belgrade, Serbia

## Abstract

Insulin resistance is believed to be an integral component of the polycystic ovary syndrome (PCOS). Beta (*ß*) cell dysfunction is also found in PCOS. In the study, we determined the influence of age, body mass index (BMI), and waist-to-hip ratio (WHR) on insulin response to oral glucose load (OGTT) and on insulin sensitivity (Si) and *ß*-cell function in young women with PCOS. One hundred fourteen patients with PCOS and 41 controls with normal basal plasma glucose were studied. A 75-g OGTT was performed to determine glucose tolerance and insulin response. Insulin sensitivity and *ß*-cell function were studied using a modified frequently sampled IV glucose tolerance test (FISGTT) to determine the acute insulin response (AIR_G_), as well as S_i_ by minimal model analysis. S_i_ was decreased in PCOS women (2.49 0.18 vs. 3.41 ± 0.36, *p* < 0.05), but no difference in AIR_G_ existed between the PCOS and control group (75.1 ± 4.6 vs. 63.4 ± 4.6, *p* < 0.05). BMI and WHR correlated negatively with S_i_ (*r* = −0.43; *r* = −0.289, *p* < 0.001, respectively), but not with AIR_G_ (*r* = 0.116; *r* = −0.02, *p* > 0.05, respectively). Increasing age correlated negatively with AIR_G_ (*r* = −0.285, *p* < 0.001). There was a significant interaction between disease (PCOS), BMI, and WHR on S_i_ as well as between age and PCOS on AIR_G_. Thus, patients below the age of 25 with PCOS showed enhanced AIR_G_ (89.5 ± 7.1 vs. 65.1 ± 6.7, *p* < 0.05) and decreased S_i_ (2.43 ± 0.25 vs. 4.52 ± 0.62, *p* < 0.05) compared to age-matched controls. In conclusion, these data suggest that not all patients with PCOS have basal and stimulated hyperinsulinemia, insulin resistance, and impaired glucose tolerance. Based on these data in young PCOS subjects, the development of insulin resistance and T2DM may be prevented with appropriate treatment strategies.

## 1. Introduction 

Polycystic ovary syndrome (PCOS) is one of the most frequent endocrinopathies in women of reproductive age [1]. The diagnosis of PCOS is suggested by the findings of hyperandrogenism and infertility [2]. Insulin resistance is a coexisting characteristic of this disorder in many mature patients with established PCOS, but its role in the pathogenesis of PCOS is unclear [3–5]. While insulin resistance may be a factor in the development of PCOS, the associated failure of pancreatic *ß*-cell function may also be an important determinant of impaired glucose tolerance or type 2 diabetes (T2DM). Knockout experiments confirm that type 2 diabetes is a “2-hit” disease, in which insulin resistance is necessarily accompanied by a *ß*-defect, preventing the compensatory up-regulation of insulin secretion [6]. The prevalence of impaired glucose tolerance (IGT) and T2DM is increased in PCOS [6]. The clinical characteristics of PCOS, including insulin resistance, have been studied in adolescent persons with this disorder. It is postulated that the disorder begins at menarche, and some characteristics change with age [7]. A previous study [8] suggested that adolescents with PCOS are severely insulin resistant, compared with a control group matched for body composition and abdominal obesity. Middle-aged PCOS women have been observed to have an increased prevalence of T2DM when compared to an age-matched control population [9].

Thus, the aim of the present study was to investigate the oral glucose tolerance test (OGTT), insulin sensitivity (S_i_), and acute insulin response (AIR_G_) during a frequently sampled glucose tolerance test (FSIGTT) as well as the interactions of age, body mass index (BMI), and waist-to-hip ratio (WHR) with the PCOS disorder in a relatively young group of women with PCOS.

## 2. Materials and Methods

### 2.1. Subjects

114 women with PCOS and 41 years of age and BMI-matched healthy women were referred consecutively to the outpatient clinic of the Clinic for Endocrinology, Diabetes, and Diseases of Metabolism for clinical hyperandrogenism and/or menstrual dysfunction. PCOS was diagnosed according to the Rotterdam workshop criteria, i.e., the presence of at least two among the three following features: clinical and/or biochemical hyperandrogenism, chronic oligoanovulation, and polycystic ovary morphology (PCOM), after exclusion of secondary causes [2]. Appropriate tests were used to confirm the absence of specific diseases of the adrenal, ovary or pituitary, such as nonclassic 21-hydroxylase deficiency, hyperprolactinemia, or androgen-secreting neoplasms [1]. No women were taking medications which could potentially interfere with the evaluations carried out in the study. Moreover, patients had not received oral contraceptives, insulin-sensitizing agents, antiandrogens, or glucocorticoids in the six months prior to the investigation. BMI was calculated as body weight/height (kg/m^2^), and WHR was determined by measuring the waist in centimeters at its smallest circumference and the hip in centimeters at the largest circumference. A BMI of 25 kg/m^2^ was determined as the borderline between overweight and nonoverweight subjects. All the investigated subjects had normal fasting plasma glucose (≤5.6 mmol/l) except one PCOS patient who had fasting plasma glucose of 5.8 mmol/L. All controls had normal glucose tolerance based on 2-hr plasma glucose levels during OGTT [10]. Women with PCOS were studied in their follicular phase of the menstrual cycle or were amenorrhoeic for more than three months, while control women were tested during their follicular phase. The local human investigation committee approved the study protocol, and all participants gave informed consent.

### 2.2. Protocol

Oral glucose tolerance test (OGTT) and intravenous glucose tolerance test (IVGTT) with frequent blood sampling (FSIGTT) were conducted on separate occasions in all subjects. Tests were performed after 3 days on a 300 g per day carbohydrate diet and after an overweight fast of 10 hr. Blood samples for plasma glucose and plasma insulin were drawn at baseline and every 30 min for 2 hr, after a 75-g glucose load. The modified IVGTT (FISGTT) was also performed after overnight fasting, according to the previously published procedures [11, 12]. After an overnight fast, catheters were placed in a forearm vein and a hand vein of the contralateral arm. Basal samples were collected for glucose and insulin at −15, −10, −5, and −1 min. Glucose (300 mg/kg) was injected as a bolus at time 0 over 1 min and flushed with saline to ensure complete delivery. After 20 min, 0.05 IU/kg of short-acting insulin (Actrapid HM, NovoNordisk) was injected. Blood samples were drawn at 2, 3, 4, 5, 6, 8, 10, 12, 14, 16, 19, 22, 23, 24, 25, 27, 30, 40, 50, 60, 70, 90, 100, 120, 140, 160, and 180 min for measuring plasma glucose and insulin levels. Glucose was measured in a Beckman glucose analyser, using the glucose oxidase method. Insulin (mU/L) and testosterone (nmol/L) (basal) were measured by radioimmunoassay (RiA INEP, Zemun).

### 2.3. Data Analysis

The area under the glucose (AUCG) and insulin (AUCI) response curves during OGTT was calculated by the standard trapezoidal rule. The insulin sensitivity index (S_i_) was calculated by a minimal model analysis using the MINMOD computer program [13]. An acute insulin response (AIR_G_) was calculated as the mean increase in insulin levels calculated from 2 to 10 min of IVGTT. The disposition index was calculated according to the following formula: DI = S_i_ × AIR. The glucose tolerance to IV glucose load (Kg) was calculated according to the standard procedures [12]. Data were compared using *T*-test, and age and BMI multivariate probability distribution was compared using the peacock test. Multivariate probability distributions were not different between the groups (*p*=0.305).

Best subset regression was done using the leaps package in order to estimate the best predictor of BMI as well as WHR [14].

Data are presented as the mean SEM. Comparisons between groups were performed using the general factorial analysis of the covariance model, controlling for the effect of BMI and WHR (ANCOVA). All analysis was done for the whole group, but some data are presented for obese and nonobese separately. All analysis was performed using SPSS and the R software package.

## 3. Results

### 3.1. Clinical Characteristics

The clinical characteristics of PCOS women and controls are presented in Table 1. There is no difference in age, BMI, WHR, fasting glucose, or blood pressure between PCOS and controls. In the PCOS group, 67.53% patients were overweight/obese. In control group 51.22% women were overweight/obese. Total testosterone levels were substantially higher (*p* < 0.001) in PCOS than in control women (3.73 ± 0.16 vs. 1.89 ± 0.10 nmol/L). As regards PCOS clinical phenotypes, 109 (95.62%) women had hyperandrogenism and polycystic ovary morphology (PCOM) and 4.38% (5 women) had hyperandrogenism and oligoanovulation.

### 3.2. Fasting Plasma Glucose, Insulin, and OGTT

**Figure 1:**
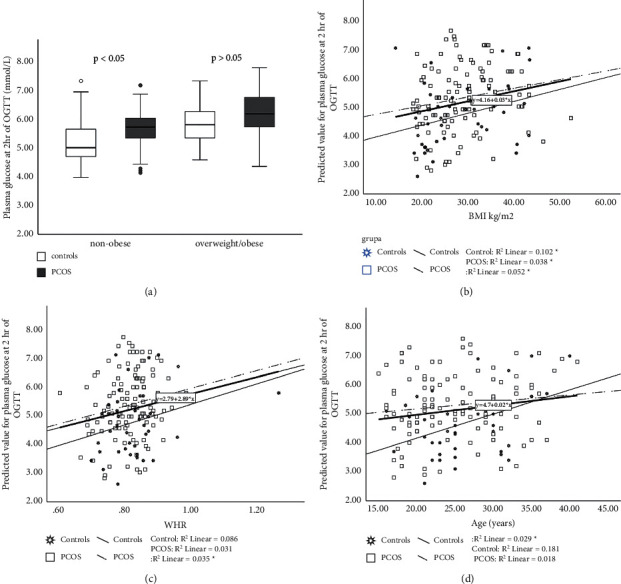
Plasma glucose at 2 hr of OGTT in PCOS patients and controls. PCOS vs. controls, *p* < 0.05. Data are presented as the mean ± SEM, separately for nonobese subjects (BMI < 25 kg/m^2^) and overweight/obese (BMI > 25 kg/m^2^) (a). Relationship between BMI and plasma glucose at 2 hr of OGTT (b). Relationship between WHR and plasma glucose at 2 hr of OGTT (c). Relationship between age and plasma glucose at 2 hr of OGTT (d). ^*∗*^*p* < 0.05.

After adjusting for BMI and using an analysis of covariance, it was observed that there was no difference in fasting plasma glucose between PCOS and controls (4.42 ± 0.06 vs. 4.37 ± 0.09 mmol/L, *p* > 0.05) (Table 2). There was a positive interaction between BMI and PCOS (*p* < 0.05). Plasma glucose at the 2 hr point of OGTT was higher in the PCOS group than in controls (*p* < 0.05) (Figure 1(a), Table 2) and in nonobese subgroup of PCOS in comparison with nonobese control (Table 3). BMI (Figure 1(b)) and WHR (Figure 1(c)) were positively correlated with plasma glucose at 2 hr of OGTT while positively correlated with this parameter only in the control group (Figure 1(d)). AUCG was not significantly higher in PCOS group vs. controls (729.47 ± 13.43 vs. 699.40 ± 24.81 mmol/L/120 min, *p* > 0.05 (Table 2)).

**Figure 2:**
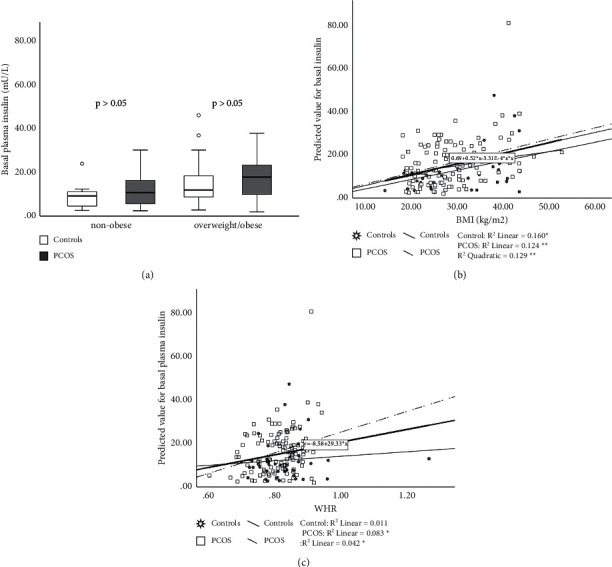
Basal plasma insulin in PCOS patients and controls. PCOS vs. controls, *p* < 0.05. Data are presented as mean ± SEM, separately for nonobese subjects (BMI < 25 kg/m^2^) and overweight/obese (BMI > 25 kg/m^2^) (a). Relationship between BMI and PCOS on basal plasma insulin (*p* < 0.001) (b). Relationship between WHR and PCOS on basal plasma insulin (*p* < 0.05) (c). ^*∗*^*p* < 0.05,  ^∗∗^*p* < 0.001.

**Figure 3:**
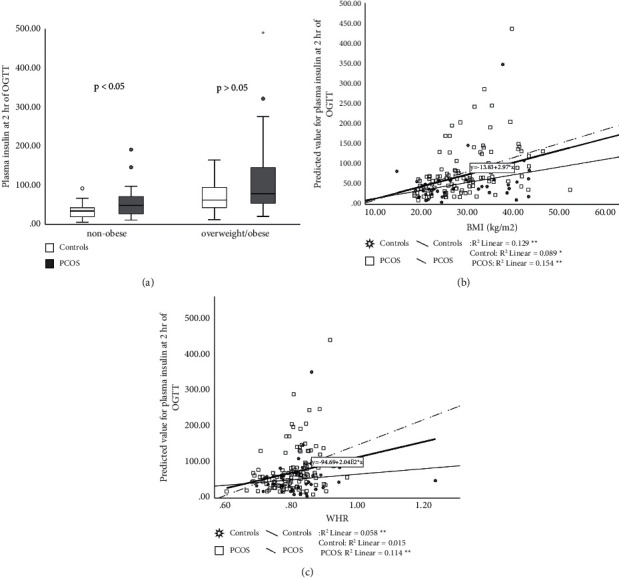
Plasma insulin at 2 hr of OGTT in PCOS patients and controls. PCOS vs. controls, *p* < 0.05. Data are presented as mean ± SEM, separately for nonobese subjects (BMI < 25 kg/m^2^) and overweight/obese (BMI > 25 kg/m^2^) (a). Relationship between BMI and PCOS on plasma insulin at 2 hr of OGTT (*p* < 0.001) (b). Relationship between WHR and PCOS on plasma insulin at 2 hr of OGTT (*p* < 0.001) (c). ^∗∗^*p* < 0.001.

Fasting plasma insulin and plasma insulin at 2 hr of OGTT were significantly higher in PCOS patients than in controls (*p* < 0.05) (Figures 2(a) and 3(a)) while AUCI was not significantly higher in PCOS group vs. controls (9931.62 ± 594.49 vs. 7816.08 ± 892.38 mU/L/120 min, *p* > 0.05) (Tables 2 and 3). BMI and WHR correlated positively with these parameters (*p* < 0.05) (Figures 2(b) and 3(b)). Significant interactions were found between PCOS and BMI on basal and stimulated plasma insulin (*p* < 0.001) (Figures 2(b) and 3(b)), as well as between WHR and PCOS on basal/stimulated insulin (*p* < 0.001, respectively) (Figures 2(c) and 3(c)).

### 3.3. Minimal Model Assessment of S_i_ and AIR_G_

**Figure 4:**
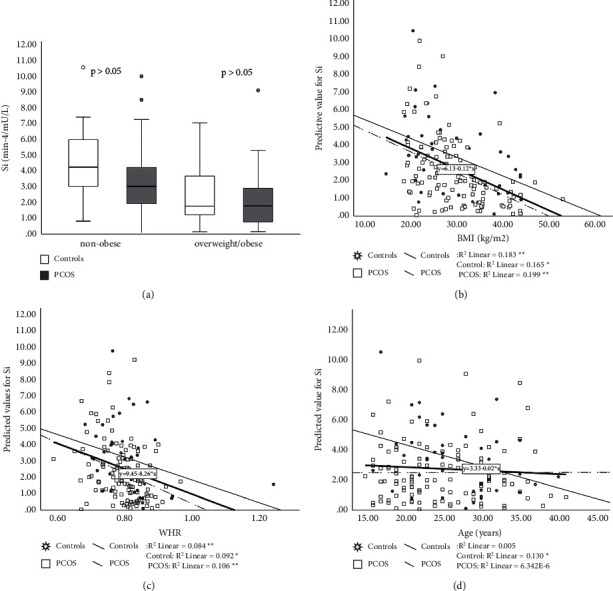
Insulin sensitivity (S_i_) in PCOS patients and controls. PCOS vs. controls, *p* < 0.05. Data are presented as mean ± SEM, separately for nonobese subjects (BMI < 25 kg/m^2^) and overweight/obese (BMI ≥ 25 kg/m^2^) (a). Relationship between BMI and PCOS on S_i_ (*p* < 0.001) (b). Relationship between WHR and PCOS on S_i_ (*p* < 0.001) (c). Relationship between age and controls on S_i_ (*p* < 0.05) (d). ^*∗*^*p* < 0.05,  ^∗∗^*p* < 0.01.

**Figure 5:**
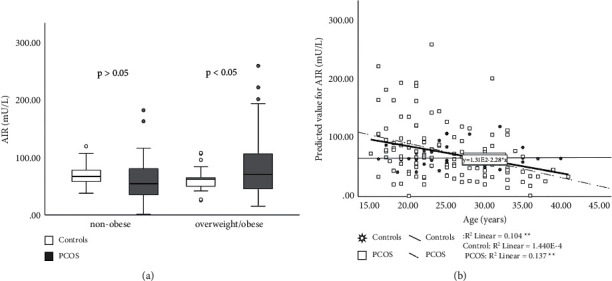
Acute insulin response during IVGTT (AIR_G_) in PCOS patients and controls. PCOS vs. controls, *p* > 0.05. Data are presented as the mean ± SEM, separately for subjects overweight/obese (BMI ≥ 25 kg/m^2^) and nonobese (BMI < 25 kg/m^2^) (a). Relationship between age and PCOS on AIR_G_ (*p* < 0.001) (b). ^∗∗^*p* < 0.01.

**Figure 6:**
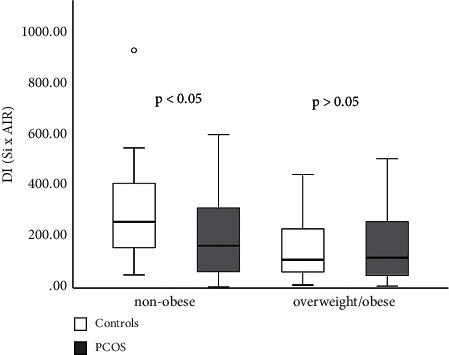
Disposition index (S_i_ × AIR_G_) in PCOS patients and controls. PCOS vs. controls, *p* > 0.05. Data are presented as the mean ± SEM, separately for nonobese subjects (BMI < 25 kg/m^2^) and overweight/obese (BMI ≥ 25 kg/m^2^).

**Figure 7:**
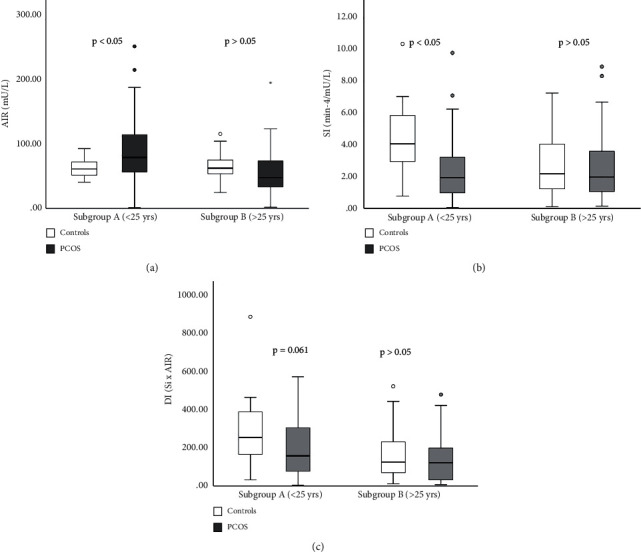
AIR_G_ in age subgroups of PCOS patients and controls. Subgroup A: <25 years old. Subgroup B: ≥25 years old. Data are presented as the mean ± SEM (a). S_i_ in age subgroups of PCOS patients and controls. Data presented as the mean ± SEM (b). DI in age subgroups of PCOS patients and controls. Data presented as the mean ± SEM (c).

S_i_ was decreased in patients with PCOS compared to controls (*p* < 0.05) (Figure 4(a)). There was a significant interaction between PCOS and BMI as well as between WHR and PCOS in relation to S_i_ (*p* < 0.001) (Figures 4(b) and 4(c)). Results obtained by measuring AIR_G_, show that AIR_G_ was not different between the PCOS and the control group (*p* > 0.05) (Figure 5(a)). With increasing age, AIR_G_ decreased in both groups. Interaction between age and PCOS on this parameter shows that AIR_G_ declines more with aging in the PCOS group (*p* < 0.001) (Figure 5(b)). Disposition index (S_i_ × AIR) was decreased in the PCOS group but not significantly compared to controls (*p* > 0.05) (Figure 6). When PCOS patients and controls were collated into two subgroups based on age (subgroup A < 25 years, subgroup B ≥ 25 years) AIR_G_ was higher in the PCOS subgroup A than in age-matched controls (*p* < 0.05) (Figure 7(a)). S_i_ was decreased in the PCOS group (*p* < 0.05) and DI was not significantly decreased in the PCOS subgroup A compared to the control subgroup A (*p*=0.061) (Figures 7(b) and 7(c)) (Table 4). The entire group of women with PCOS had a normal glucose tolerance during IVGTT (Kg), not different from controls (2.01 ± 0.11 vs. 1.88 ± 0.14 × 10^2^, *p* > 0.05). A positive relationship between BMI and WHR with PCOS was also confirmed (*p* < 0.05). The best predictors of BMI as well as WHR were presented in Table 5.

## 4. Discussion

The prevalence of impaired glucose tolerance (IGT) and diabetes increased in PCOS [14, 15]. Our data confirmed that plasma glucose at the 2 hr point of OGTT was higher in the PCOS group than in controls although all investigated subjects had normal fasting glucose and normal glucose tolerance. The PCOS subjects in this investigation had higher basal plasma insulin and higher plasma insulin levels at 2 hr of OGTT compared to controls. Our results agree with the notion that high BMI and central obesity cause exaggerated insulin responses in PCOS women [16, 17]. Insulin resistance occurs in 40%–70% of women with PCOS [18]. Obesity may increase PCOS prevalence and exacerbate IR in women with PCOS [19], while insulin resistance in lean women with PCOS is not consistently demonstrated [20]. The euglycemic-hyperinsulinemic clamp is the gold standard to directly measure insulin sensitivity. In some previous studies [21, 22] it was showed that women with PCOS have intrinsic reduction in insulin sensitivity on euglycemic‐hyperinsulinemic clamp and almost all obese women with PCOS have more serious IR than lean women with PCOS. A systematic review and meta‐analysis of euglycemic‐hyperinsulinemic clamp studies by Cassar et al. showed a reduction in insulin sensitivity of 27% and obesity exacerbates the reduction in insulin sensitivity by 15% in women with PCOS [23]. Our results confirmed the existence of insulin resistance in young PCOS patients. After adjusting for BMI, S_i_ remained lower in PCOS women than in controls. Overweight contributed to the impairment of insulin sensitivity in PCOS as well as controls [24]. Furthermore, our results showed that the interaction between disease (PCOS) and being overweight exists. These data suggest that increased BMI may have a more deleterious effect on insulin sensitivity in PCOS than in controls. This is important because overweight or obesity is detected in about 30–50% women with PCOS [9]. Furthermore, our data indicate that individual lean patients with PCOS often have no hyperinsulinemia and insulin resistance as it was shown in previous study [25].

Android obesity, clinically confirmed as increased WHR, is an especially strong risk for insulin resistance and other factors that predispose to premature cardiovascular disease [20]. Although we were unable to demonstrate a difference in WHR between PCOS and healthy women, our results confirmed a significant influence of android obesity towards insulin resistance in both PCOS and controls. Thus, our data also indicated that PCOS women are more susceptible to increasing WHR regarding the development of insulin resistance.

The product of insulin sensitivity (Si) and insulin response (AIR_G_) in healthy subjects is a constant value that has been termed the disposition index (DI) [12]. It is expected that AIR_G_ will be higher as a compensatory mechanism in the state of insulin resistance [13]. On the other hand, the DI is low in subjects with IGT or T2DM [25]. In our study, we demonstrated decreased DI in nonobese PCOS compared to controls. A possible explanation for this finding is the lack of a compensatory insulin response in nonobese PCOS women as would be expected in an insulin resistant state.

Interestingly, the data show that there is no difference in AIR_G_ between PCOS and controls. Results from this study clearly show a significant negative correlation between AIR_G_ and increasing age. With an increase in age, AIR_G_ falls faster in patients with PCOS. Interestingly, our data did not confirm the significant influence of obesity on this parameter [24].

While insulin resistance is a factor in the development of PCOS, the associated failure of pancreatic *ß*-cell function could be an important determinant of the development of T2DM in many of these women [26–28]. Although most of the observed hyperinsulinemia in PCOS is probably secondary to insulin resistance, there seems to be an important component of abnormal insulin secretion, which is independent of insulin resistance, body weight, and body fat distribution [3, 8, 29, 30]. Thus, besides insulin resistance, *ß*-cell dysfunction seems to be an integral characteristic of this syndrome [27]. A recent study showed that in women with PCOS, metabolic clearance of insulin is reduced, contributing to developing hyperinsulinemia, as well as that serum androgens are independent predictors of this phenomenon [28]. But it was demonstrated that a higher prevalence of impaired insulin secretion than impaired insulin action exists in first-degree relatives of patients with PCOS [27, 31].

According to a previously published report [26], *ß*-cell secretory defects may contribute to increased carbohydrate intake and subsequently obesity and insulin resistance. Current data support the notion that glucose intolerance and frank T2DM are found in combination with the sign of *ß*-cell exhaustion in a significant portion of young women with PCOS. These subjects with PCOS may manifest later stage carbohydrate intolerance and T2DM after long-standing insulin resistance in susceptible women [26]. In this regard, our data could suggest that an intrinsic defect in insulin secretion exists in PCOS patients.

In the youngest PCOS women (subgroup A), we demonstrated exaggerated AIR_G_ in comparison with age-matched controls as well as compared to older PCOS women (subgroup B). The exaggerated AIR_G_ in our young patients with PCOS could be a compensatory response to underlying insulin resistance. Despite the compensatory response, this group failed to achieve a DI observed in controls.

Current data are in concert with prior reports that significant abnormalities in insulin secretion are already present in patients with PCOS <25 years [31, 32]. Although, in our younger PCOS women, AIR_G_ was enhanced, the DI was still decreased compared to age-matched controls due to significantly impaired S_i_, placing these patients at heightened risk for T2DM. Studies on young adolescent girls may provide clues about the pathophysiology of PCOS since this is an age when early clinical signs are manifested. Thus, this may be the time to initiate hygienic and medical interventions to retard the development of impaired glucose intolerance and T2DM [27, 29–31, 33]. This study suggests that young PCOS women can indeed be identified and placed on therapy to reduce the cardiovascular risk factors and development of T2DM [10, 34–49].

## 5. Conclusion

The major strength of this study is the large number of PCOS women investigated using minimal model analyses to evaluate acute insulin response and assess insulin secretion as well as insulin sensitivity. However, there are also limitations in the study. This is an observational study, and cause-effect relationships cannot be firmly established. In addition, as most women in this cohort had a hyperandrogenic PCOS phenotype, the results of the study may be more applicable to these subjects.

In conclusion, current observations underlie the importance of interactions between PCOS, BMI, age, and WHR. This investigation has also shown that not all patients with PCOS demonstrate basal and stimulated hyperinsulinemia, insulin resistance, and impaired glucose tolerance, particularly early in the evolution of PCOS as a clinical entity. Our data concerning subjects younger than 25 years underscores the importance of establishing the diagnosis of PCOS in adolescence, and the institution of appropriate therapy targeting insulin resistance and *ß*-cell secretion before T2DM develops.

## Figures and Tables

**Table 1:** Characteristics of studied PCOS women and controls (mean ± SEM).

Characteristics	PCOS (*n* = 114)	Controls (*n* = 41)
	(Mean ± SE)	(Mean ± SE)
AGE (years)	24.88 ± 0.59	26.60 ± 0.89
BMI (kg/m^2^)	28.80 ± 0.67	28.55 ± 1.35
WHR	0.81 ± 0.006	0.82 ± 0.015
Fasting plasma glucose (mmol/L)	4.42 ± 0.06	4.37 ± 0.09
Total testosterone (nmol/L)^*∗*^	3.73 ± 0.16	1.89 ± 0.10
Systolic BP (mmHg)	125.12 ± 1.13	126.39 ± 2.30
Diastolic BP (mmHg)	80.61 ± 0.93	82.92 ± 1.85

^*∗*^ *p* < 0.05.

**Table 2:** Investigated indices in PCOS and controls.

Parameter	PCOS	Controls	*P* value
Fasting glucose (mmol/L)	4.42 ± 0.06	4.37 ± 0.089	*p* > 0.05
Plasma glucose at 120 min of OGTT (mmol/L)	5.29 ± 0.10	4.72 ± 0.17	*p* < 0.05
Fasting insulin (mU/L)	16.35 ± 0.99	12.34 ± 1.47	*p* < 0.05
Plasma insulin at 120 min of OGTT (mU/L)	78.52 ± 5.93	52.07 ± 8.69	*p* < 0.05
Area under glucose curve (OGTT)	729.47 ± 13.43	699.40 ± 24.81	*p* > 0.05
Area under insulin curve (OGTT)	9931.62 ± 594.49	7816.08 ± 892.38	*p* < 0.05
Si (insulin sensitivity, minimal model analysis)	2.49 ± 0.18	3.41 ± 0.36	*p* < 0.05
AIR (acute insulin response, minimal model analysis)	76.29 ± 4.56	65.69 ± 3.28	*p* > 0.05
Di (disposition index, minimal model analysis)	171.07 ± 13.07	220.28 ± 28.12	*p* > 0.05

**Table 3:** Investigated indices in PCOS and controls (nonobese vs. overweight/obese).

Parameter	PCOS vs. controls (nonobese)	*P* value	PCOS vs. controls (overweight/obese)	*P* value
Fasting glucose (mmol/L)	4.34 ± 0.11 vs. 4.14 ± 0.13	*p* > 0.05	4.46 ± 0.08 vs. 4.58 ± 0.10	*p* > 0.05
Plasma glucose at 120 min of OGTT (mmol/L)	4.87 ± 0.16 vs. 4.26 ± 0.23	*p* < 0.05	5.50 ± 0.13 vs. 5.10 ± 0.21	*p* > 0.05
Fasting insulin (mU/L)	12.33 ± 1.35 vs. 8.93 ± 1.09	*p* > 0.05	18.351.28 vs. 15.58±±2.51	*p* > 0.05
Plasma insulin at 120 min of OGTT (mU/L)	48.19 ± 5.13 vs. 31.50 ± 4.29	*p* < 0.05	93.68 ± 7.99 vs. 71.65 ± 15.45	*p* > 0.05
Area under glucose curve (OGTT)	669.28 ± 21.58 vs. 651.53 ± 37.89	*p* > 0.05	759.57 ± 16.01 vs. 744.99 ± 29.86	*p* > 0.05
Area under insulin curve (OGTT)	7163.16 ± 852.17 vs. 5681.55 ± 460.25	*p* > 0.05	11315.86 ± 736.86 vs. 9848.96 ± 1579.33	*p* > 0.05
Si (insulin sensitivity, minimal model analysis)	3.39 ± 0.38 vs.4.48 ± 0.52	*p* > 0.05	2.03 ± 0.39 vs. 2.40 ± 0.39	*p* < 0.05
AIR (acute insulin response, minimal model analysis)	61.78 ± 6.12vs. 69.37 ± 4.47	*p* > 0.05	83.56 ± 5.96 vs. 62.21 ± 4.76	*p* > 0.05
Di (disposition index, minimal model analysis)	190.29 ± 24.16 vs. 298.05 ± 45.58	*p* < 0.05	161.45 ± 15.44 vs. 146.22 ± 25.40	*p* > 0.05

**Table 4:** Investigated indices in PCOS and controls (<25 years old vs. ≥25 years old).

Parameter	PCOS vs. controls (<25 years old)	*P* value	PCOS vs. controls (≥25 years old)	*P* value
Fasting glucose (mmol/L)	4.39 ± 0.09 vs. 4.33 ± 0.18	*p* > 0.05	4.45 ± 0.09 vs. 4.39 ± 0.09	*p* > 0.05
Plasma glucose at 120 min of OGTT (mmol/L)	5.16 ± 0.14 vs. 4.36 ± 0.23	*p* < 0.05	5.44 ± 0.15 vs. 4.94 ± 0.24	*p* > 0.05
Fasting insulin (mU/L)	16.09 ± 1.18 vs. 14.02 ± 3.20	*p* > 0.05	16.63 ± 1.66 vs. 11.26 ± 1.29	*p* < 0.05
Plasma insulin at 120 min of OGTT (mU/L)	86.30 ± 9,82 vs. 42.42 ± 6.49	*p* < 0.05	69.87 ± 6.04 vs. 58.24 ± 13.61	*p* > 0.05
Area under glucose curve (OGTT)	704.59 ± 18.19 vs. 665.72 ± 32.78	*p* > 0.05	757.12 ± 19.36 vs. 720.96 ± 34.69	*p* > 0.05
Area under insulin curve (OGTT)	10508.03 ± 934.95 vs. 7000.97 ± 834.59	*p*=0.061	9291.17 ± 703.45 vs. 8337.75 ± 1367.11	*p* > 0.05
Si (insulin sensitivity, minimal model analysis)	2.43 ± 0.25 vs. 4.52 ± 0.62	*p* < 0.05	2.54 ± 0.27 vs. 2.71 ± 0.38	*p* > 0.05
AIR (acute insulin response, minimal model analysis)	92.57 ± 6.88 vs. 64.14 ± 3.88	*p* < 0.05	58.22 ± 4.82 vs. 66.69 ± 4.82	*p* > 0.05
Di (disposition index, minimal model analysis)	195.77 ± 18.89 vs. 308.23 ± 53.15	*p* > 0.05	143.61 ± 17.34 vs. 163.99 ± 26.33	*p* > 0.05

**Table 5:** Regression analysis: best predictors of BMI and WHR.

	Estimate	Std. error	Coefficient	*p* value
BMI				
Intercept	24.12239	2.48639		
Age	0.20362	0.08492		
Basal insulin	0.19604	0.05077		
Si	−1.29620	0.25430		
			*r* = 0.521	<0.001

WHR				
Intercept	0.67519190	0.03245457	20.804	
Age	0.00352968	0.00088745	3.977	
AUCG	0.00009734	0.00003786	2.571	
Si	−0.00778025	0.00255817	−3.041	
			*r* = 0.474	<0.001

## Data Availability

Data can be made available on reasonable request (msumaracdumanovic@gmail.com).

## References

[1] Franks S. (1995). Polycystic ovary syndrome. *New England Journal of Medicine*.

[2] The Rotterdam ESHRE/ASRM-Sponsored PCOS Consensus Workshop Group (2004). Revised 2003 consensus on diagnostic criteria and long-term health risks related to polycystic ovary syndrome (PCOS). *Human Reproduction*.

[3] Johnstone E. B., Rosen M. P., Neril R. (2010). The polycystic ovary post-Rotterdam: a common, age-dependent finding in ovulatory women without metabolic significance. *Journal of Clinical Endocrinology & Metabolism*.

[4] Nestler J. E. (1997). Role of hyperinsulinemia in the pathogenesis of the polycystic ovary syndrome, and its clinical implications. *Seminars in Reproductive Endocrinology*.

[5] O’Meara N. M., Blackman J. D., Ehrmann D. A. (1993). Defects in beta-cell function in functional ovarian hyperandrogenism. *Journal of Clinical Endocrinology & Metabolism*.

[6] Legro R. S., Kunselman A. R., Dodson W. C., Dunaif A. (1999). Prevalence and predictors of risk for type 2 diabetes mellitus and impaired glucose tolerance in polycystic ovary syndrome: a prospective, controlled study in 254 affected women. *Journal of Clinical Endocrinology & Metabolism*.

[7] Bili H., Laven J., Imani B., Eijkemans M. J., Fauser B. C. (2001). Age-related differences in features associated with polycystic ovary syndrome in normogonadotrophic oligo-amenorrhoeic infertile women of reproductive years. *European Journal of Endocrinology*.

[8] Arslanian S. A., Lewy V. D., Danadian K. (2001). Glucose intolerance in obese adolescents with polycystic ovary syndrome: roles of insulin resistance and *β*-cell dysfunction and risk of cardiovascular disease. *Journal of Clinical Endocrinology & Metabolism*.

[9] Diamanti-Kandarakis E., Dunaif A. (2012). Insulin resistance and the polycystic ovary syndrome revisited: an update on mechanisms and implications. *Endocrine Reviews*.

[10] World Health Organization Study Group (1985). Diabetes mellitus. *World Health Organization Technical Report Series*.

[11] Bergman R. N., Ider Y. Z., Bowden C. R., Cobelli C. (1979). Quantitative estimation of insulin sensitivity. *American Journal of Physiology, Endocrinology and Metabolism*.

[12] Pacini G., Bergman R. N. (1986). MINMOD: a computer program to calculate insulin sensitivity and pancreatic responsivity from the frequently sampled intravenous glucose tolerance test. *Computer Methods and Programs in Biomedicine*.

[13] Bergman R. N., Finegood D. T., Kahn S. E. (2002). The evolution of *β*‐cell dysfunction and insulin resistance in type 2 diabetes. *European Journal of Clinical Investigation*.

[14] Miller A. (2020). *Leaps: Regression Subset Selection. R package version 3.1*.

[15] Conn J. J., Jacobs H. S., Conway G. S. (2000). The prevalence of polycystic ovaries in women with type 2 diabetes mellitus. *Clinical Endocrinology*.

[16] Pasquali R., Casimirri F., Venturoli S. (1994). Body fat distribution has weight-independent effects on clinical, hormonal, and metabolic features of women with polycystic ovary syndrome. *Metabolism*.

[17] Ovalle F., Azziz R. (2002). Insulin resistance, polycystic ovary syndrome, and type 2 diabetes mellitus. *Fertility and Sterility*.

[18] DeUgarte C. M., Bartolucci A., Azziz A. (2005). Prevalence of insulin resistance in the polycystic ovary syndrome using the homeostasis model assessment. *Fertility and Sterility*.

[19] Teede H., Deeks A., Moran L. (2010). Polycystic ovary syndrome: a complex condition with psychological, reproductive and metabolic manifestations that impacts on health across the lifespan. *BMC Medicine*.

[20] Gennarelli G., Rovei V., Novi R. F. (2005). Preserved insulin sensitivity and *β*-cell activity, but decreased glucose effectiveness in normal-weight women with the polycystic ovary syndrome. *Journal of Clinical Endocrinology & Metabolism*.

[21] Stepto N. K., Cassar S., Joham A. E. (2013). Women with polycystic ovary syndrome have intrinsic insulin resistance on euglycaemic‐hyperinsulaemic clamp. *Human Reproduction*.

[22] Tosi F., Bonora E., Moghetti P. (2017). Insulin resistance in a large cohort of women with polycystic ovary syndrome: a comparison between euglycaemic‐hyperinsulinaemic clamp and surrogate indexes. *Human Reproduction*.

[23] Cassar S., Misso M. L., Hopkins W. G., Shaw C. S., Teede H. J., Stepto N. K. (2016). Insulin resistance in polycystic ovary syndrome: a systematic review and meta‐analysis of euglycaemic‐hyperinsulinaemic clamp studies. *Human Reproduction*.

[24] Dunaif A., Finegood D. T. (1996). Beta-cell dysfunction independent of obesity and glucose intolerance in the polycystic ovary syndrome. *Journal of Clinical Endocrinology & Metabolism*.

[25] Ovesen P., Moller J. E. N. S., Ingerslev H. J. (1993). Normal basal and insulin-stimulated fuel metabolism in lean women with the polycystic ovary syndrome. *Journal of Clinical Endocrinology & Metabolism*.

[26] Holte J., Bergh T., Berne C., Berglund L., Lithell H. (1994). Enhanced early insulin response to glucose in relation to insulin resistance in women with polycystic ovary syndrome and normal glucose tolerance. *Journal of Clinical Endocrinology & Metabolism*.

[27] Cavaghan M. K., Ehrmann D. A., Polonsky K. S. (2000). Interactions between insulin resistance and insulin secretion in the development of glucose intolerance. *Journal of Clinical Investigation*.

[28] Tosi F., Dal Molin F., Zamboni F. (2020). Serum androgens are independent predictors of insulin clearance but not of insulin secretion in women with pcos. *Journal of Clinical Endocrinology & Metabolism*.

[29] Holte J., Bergh T., Berne C. H., Wide L., Lithell H. (1995). Restored insulin sensitivity but persistently increased early insulin secretion after weight loss in obese women with polycystic ovary syndrome. *Journal of Clinical Endocrinology & Metabolism*.

[30] Holte J. (1996). Disturbances in insulin secretion and sensitivity in women with the polycystic ovary syndrome. *Baillière’s Clinical Endocrinology and Metabolism*.

[31] Colilla S., Cox N. J., Ehrmann D. A. (2001). Heritability of insulin secretion and insulin action in women with polycystic ovary syndrome and their first-degree relatives. *Journal of Clinical Endocrinology & Metabolism*.

[32] Dunaif A., Thomas A. (2001). Current concepts in the polycystic ovary syndrome. *Annual Review of Medicine*.

[33] Chang R. J., Nakamura R. M., Judd H. L., Kaplan S. A. (1983). Insulin resistance in nonobese patients with polycystic ovarian disease. *Journal of Clinical Endocrinology & Metabolism*.

[34] Cataldo N. A., Abbasi F., McLaughlin T. L., Lamendola C., Reaven G. M. (2001). Improvement in insulin sensitivity followed by ovulation and pregnancy in a woman with polycystic ovary syndrome who was treated with rosiglitazone. *Fertility and Sterility*.

[35] Palmert M. R., Gordon C. M., Kartashov A. I., Legro R. S., Emans S. J., Dunaif A. (2002). Screening for abnormal glucose tolerance in adolescents with polycystic ovary syndrome. *Journal of Clinical Endocrinology & Metabolism*.

[36] Legro R. S. (2002). Polycystic ovary syndrome long term sequelae and management. *Minerva Ginecologica*.

[37] Macut D., Micić D., Cvijović G. (2001). Cardiovascular risk in adolescent and young adult obese females with polycystic ovary syndrome (PCOS). *Journal of Pediatric Endocrinology & Metabolism*.

[38] Wild S., Pierpoint T., Jacobs H., McKeigue P. (2000). Long-term consequences of polycystic ovary syndrome: results of a 31-year follow-up study. *Human Fertility*.

[39] Nestler J. E., Stovall D., Akhter N., Iuorno M. J., Jakubowicz D. J. (2002). Strategies for the use of insulin-sensitizing drugs to treat infertility in women with polycystic ovary syndrome. *Fertility and Sterility*.

[40] Pierpoint T., McKeigue P. M., Isaacs A. J., Wild S. H., Jacobs H. S. (1998). Mortality of women with polycystic ovary syndrome at long-term follow-up. *Journal of Clinical Epidemiology*.

[41] Arslanian S. A., Lewy V., Danadian K., Saad R. (2002). Metformin therapy in obese adolescents with polycystic ovary syndrome and impaired glucose tolerance: amelioration of exaggerated adrenal response to adrenocorticotropin with reduction of insulinemia/insulin resistance. *Journal of Clinical Endocrinology & Metabolism*.

[42] Homburg R. (2002). Should patients with polycystic ovarian syndrome be treated with metformin? A note of cautious optimism. *Human Reproduction*.

[43] Marshall J. C., Dunaif A. (2012). Аll women with PCOS should be treated for insulin resistance. *Fertility and Sterility*.

[44] McFarlane S. I., Banerji M., Sowers J. R. (2001). Insulin resistance and cardiovascular Disease1. *Journal of Clinical Endocrinology & Metabolism*.

[45] Wang P., Streicher P., Glueck C. J. (2002). Treatment of polycystic ovary syndrome with insulin-lowering agents. *Expert Opinion on Pharmacotherapy*.

[46] Nestler J. E. (2002). Should patients with polycystic ovarian syndrome be treated with metformin? An enthusiastic endorsement. *Human Reproduction*.

[47] Franks S. (2002). Adult polycystic ovary syndrome begins in childhood. *Best Practice & Research Clinical Endocrinology & Metabolism*.

[48] Michelmore K. F. (2000). Polycystic ovary syndrome in adolescence and early adulthood. *Human Fertility*.

[49] Abbott D. H., Dumesic D. A., Franks S. (2002). Developmental origin of polycystic ovary syndrome-a hypothesis. *Journal of Endocrinology*.

